# Guided, internet-based, rumination-focused cognitive behavioural therapy (i-RFCBT) versus a no-intervention control to prevent depression in high-ruminating young adults, along with an adjunct assessment of the feasibility of unguided i-RFCBT, in the REducing Stress and Preventing Depression trial (RESPOND): study protocol for a phase III randomised controlled trial

**DOI:** 10.1186/s13063-015-1128-9

**Published:** 2016-01-04

**Authors:** Lorna Cook, Edward Watkins

**Affiliations:** Mood Disorders Centre, School of Psychology, University of Exeter, Exeter, EX4 4QG UK

**Keywords:** Randomised controlled trial, Cognitive behavioural therapy (CBT), Rumination, Depression, Prevention, Internet delivery

## Abstract

**Background:**

Depression is a global health challenge. Prevention is highlighted as a priority to reduce its prevalence. Although effective preventive interventions exist, the efficacy and coverage can be improved. One proposed means to increase efficacy is by using interventions to target specific risk factors, such as rumination. Rumination-focused CBT (RFCBT) was developed to specifically target depressive rumination and reduces acute depressive symptoms and relapse for patients with residual depression in a randomised controlled trial. Preliminary findings from a Dutch randomised prevention trial in 251 high-risk 15- to 22-year-old subjects selected with elevated worry and rumination found that both supported internet-RFBCT and group-delivered RFCBT equally reduced depressive symptoms and the onset of depressive cases over a period of 1 year, relative to the no-intervention control.

**Methods/Design:**

A phase III randomised controlled trial following the Medical Research Council (MRC) Complex Interventions Framework will extend a Dutch trial to the United Kingdom, with the addition of diagnostic interviews, primarily to test whether guided internet-RFCBT reduces the onset of depression relative to a no-intervention control. High-risk young adults (aged 18 to 24 years), selected with elevated worry/rumination and recruited through university and internet advertisement, will be randomised to receive either guided internet-RFCBT, supported by clinical psychologists or mental health paraprofessionals, or a no-intervention control. As an adjunct arm, participants are also randomised to unguided internet-RFCBT self-help to provide an initial test of the feasibility and effect size of this intervention. While participants are also randomised to unguided internet-RFCBT, the trial was designed and powered as a phase III trial comparing guided internet-RFCBT versus a no-intervention control. In the comparison between these two arms, the primary outcomes are as follows: a) onset of major depressive episode over a 12-month period, assessed with a Structured Clinical Interview for Diagnosis at 3 months (post-intervention), 6 months and 15 months after randomisation. The following secondary outcomes will be recorded: the incidence of generalized anxiety disorder, symptoms of depression and anxiety, and levels of worry and rumination, measured at baseline and at the same follow-up intervals. In relation to the pilot investigation of unguided internet-RFCBT (the adjunct intervention arm), we will assess the feasibility and acceptability of the data-collection procedures, levels of attrition, effect size and acceptability of the unguided internet-RFCBT intervention.

**Discussion:**

Widespread implementation is necessary for effective prevention, suggesting that the internet may be a valuable mode of delivery. Previous research suggests that guided internet-RFCBT reduces incidence rates relative to controls. We are also interested in developing and evaluating an unguided version to potentially increase the availability and reduce the costs.

**Trial Registration:**

Current Controlled Trials ISRCTN12683436. Date of registration: 27 October 2014

**Electronic supplementary material:**

The online version of this article (doi:10.1186/s13063-015-1128-9) contains supplementary material, which is available to authorized users.

## Background

Depression is one of the leading causes of disease burden worldwide, with substantial individual, societal and economic impact [1]. Current treatments have only a limited impact on this disease burden because of the lack of treatment availability, the high proportion of patients failing to respond (40 %, including partial and non-response) and the high levels of relapse or recurrence (50-80 %) [2]. In addition, treatments provided in the acute phase of the disorder do not reduce the incidence rates. Effective preventive interventions are therefore needed to reduce the disease burden of depression.

Preventive interventions for depression have mainly focused on children and adolescents and have predominantly used CBT strategies. Meta-analyses [3–5] have found that universal interventions (aimed at entire populations, regardless of risk factors) had only small-to-insignificant effect sizes post-intervention and longer-term effects were mixed. Greater effect sizes were found for targeted interventions (selective: aimed at a subgroup presenting with known risk factors; indicated: aimed at individuals with subclinical symptoms), and these effects were sustained for longer than universal interventions [5]. Critiques of prevention research trials include a focus on treatment effects (change in symptoms relative to controls [3]) rather than on a reduction in incidence rates [6]; insufficient statistical power due to low base-rates (particularly in universal samples); and short follow-up periods, which interfere with the elucidation of longer-term effects [5].

While there are some early positive findings from prevention research, improvement is possible in terms of efficacy, cost-effectiveness and acceptability [7]. First, interventions should be targeted at higher-risk individuals rather than at universal populations [7]. Such individuals have the most to gain from an intervention, and the base rate of new cases will be higher in high-risk groups, meaning that smaller sample sizes are needed to obtain adequate statistical power to detect an effect [6]. Such individuals could be identified on the basis of personal and familial history, cognitive or biological vulnerabilities to depression, and/or subsyndromal symptoms. Risk factors can be used not only to identify individuals but also as a direct target for intervention [7]. Additionally, although current interventions are largely aimed at specific disorders, evidence is increasing for transdiagnostic processes, which are risk factors for multiple disorders [8, 9]. Targeting such processes has the potential to impact several disorders with a single intervention [10].

Repetitive negative thought (RNT), incorporating worry and rumination, has been proposed as a potential target for selective prevention programmes [7]. Strong evidence exists that RNT contributes to the onset and maintenance of a range of disorders, including depression, anxiety and physical health issues [11]. Prospective longitudinal studies have found that rumination predicted a) future depressive symptoms, even after controlling for baseline depressive and anxious symptoms, across a range of follow-up periods [12–15]; b) onset and duration of episodes of major depression [16–19]; and c) mediated the effects of other risk factors on the onset of depression [20]. Rumination and worry have also been found to predict future levels of other disorders, including anxiety, eating disorders and substance abuse [18]. Furthermore, experimental studies provide evidence that rumination causally influences negative mood and a range of cognitive processes [11], including increased negative thinking [21], impaired concentration [22] and impaired problem-solving [23]. This relationship between RNT and emotional disorders is also found in children and adolescents [12, 18, 24–26]. Moreover, Hankin [27] demonstrated that rumination is a specific risk factor for depression in adolescence, with rumination prospectively predicting fluctuations in depressive symptoms and internalising problems, but not anxious arousal or externalising problems. A gender bias exists in depression rates (approximately 2:1 female: male [28]). Differences in rumination levels can partially explain this gender bias, with 27 % of the association between gender and depression explained by rumination [27]. Critically, recent experimental and clinical studies have shown that with brief interventions, levels of rumination and worry can be reduced (e.g. [29–33]). This suggests that rumination/worry could be a particularly promising target in improving the efficacy of preventive interventions.

Widespread dissemination and coverage of interventions is important for effective prevention. Traditional face-to-face therapy is not well-suited for such widespread implementation. Alternative modes of delivery, such as internet-based treatments, have potential advantages including the following: reaching large numbers at a relatively low cost; greater ease of access and convenience for the user; available at any time and place; and greater autonomy for the client, anonymity and greater time to reflect [6, 34, 35]. Emergent evidence exists that internet-based CBT is an effective, feasible and acceptable treatment for acute depression and anxiety [36, 37]. Acceptability ratings for internet-based therapies are generally high [35]. However, difficulties with motivation and adherence are a potentially important issue for Internet-based therapies for depression. Although purely self-guided internet interventions for depression are available, they are generally less effective and have greater drop-out than guided interventions for depression with therapist input [36, 38, 39]. Andersson [34] therefore argued for some form of guidance in order to achieve similar effectiveness to face-to-face therapies.

The feasibility, acceptability, and efficacy of internet-based rumination-focused CBT (i-RFCBT) to prevent depression in adolescents and young adults has recently been investigated in the Netherlands (Topper, Emmelkamp, Watkins and Ehring: Prevention of anxiety disorders and depression by targeting excessive worry and rumination in adolescents and young adults; A randomized controlled trial. Submitted; ZonMw funded project). The intervention is based on rumination-focused cognitive behavioural therapy (RFCBT) developed by Watkins et al. [30, 32]. Watkins et al. [32] conducted a randomised-controlled trial comparing treatment-as-usual (TAU, antidepressant medication) versus TAU plus RFCBT in patients with medication-refractory residual depression. The addition of RFCBT reduced depressive symptoms more than medication alone, with improvements in remission rates (62 % vs. 21 %) and reduction in 5-month relapse rates (9.5 % vs. 53 %) Moreover, changes in rumination scores were found to be a significant mediator of these treatment effects. These positive treatment and relapse prevention results led Topper et al. to adapt this intervention for prevention.

In a prevention trial of RFCBT, 251 students aged 15-22 with elevated worry and/or rumination (scoring on top quartiles of respective measures: ≥ 50 on Penn State Worry Questionnaire (PSWQ; [40]; ≥ 40 on Ruminative Response Scale of the Response Styles Questionnaire (RRS; [15])) but who did not currently meet diagnostic criteria for depression or generalized anxiety disorder were recruited, and randomised into face-to-face group RFCBT, internet-delivered RFCBT (i-RFCBT), or a no-intervention control group. Participants were assessed for symptoms and ‘caseness’ of depression and anxiety on standardised self-report measures (Patient Health Questionnaire, PHQ-9; [41]; Generalized Anxiety Disorder Screener, GAD-7; [42]) at baseline, post-intervention, 3 month and 12 month follow-ups. This study indicated the feasibility of recruiting and retaining participants for such a prevention study and indicated the acceptability of these interventions. Further, group RFCBT and internet RFCBT reduced worry, rumination, depression and anxiety symptoms significantly more than waiting list control. At 12 month follow-up, cumulative incidence rates for depression (estimated from “caseness” on PHQ-9) are reduced in the two RFCBT intervention groups (13.1 %) relative to controls (32.2 %). Moreover, reductions in rumination and worry were found to mediate the effects of RFCBT on symptom severity.

These findings are encouraging. However, there are several key limitations of the Dutch study: (a) there is not a diagnostic interview to determine incidence of major depression, with the study instead using self-report and only able to assess point prevalence rather than retrospective incidence; (b) similarly, it is not known whether the participants had previous episodes of depression, i.e., whether the intervention prevents first onset or relapse/recurrence of depression.

### Study aims and objectives

The primary aim of this phase III efficacy trial is to replicate and extend the Dutch study to compare guided i-RFCBT with a no-intervention control. If the target sample size based on the comparison of guided i-RFCBT to controls in the Dutch trial is achieved, the aim is to assess whether the findings from this previous trial can be replicated in a different population of undergraduates in the United Kingdom.

The RESPOND study will address the limitations of the Dutch trial by including a well-validated diagnostic interview (Structured Clinical Interview for DSM-IV (SCID-I [43]) to increase accuracy in determining current and past diagnostic status, and to allow for stratification on history of depression. Although including previously depressed participants is not consistent with strict definitions of initial onset prevention studies, there is a strong argument for preventing further relapses and recurrences, especially as a major risk factor for a depressive episode is a history of depressive episodes [6].

One key change from the design of the Dutch trial is the selection of a young adult population aged 18-24-years, principally undergraduates, rather than 15-22 years-old, in order to recruit participants able to provide their own informed consent. This age group was chosen for a number of reasons: (a) for pragmatic reasons to improve feasibility and logistics of recruitment, since parental consent can be a barrier to recruitment for an internet-based prevention study [44]; (b) larger effect sizes are found for older adolescents in preventive studies, perhaps because older adolescents are better able to understand the intervention compared to younger adolescents or children [4] and c) life transitions such as moving out of the family home have been shown to increase the incidence of depression [45]. Additionally depression is prevalent in this population [46] and the number of students experiencing high levels of worry, stress and symptoms of depression and anxiety is also high [47], indicating that there is a significant group of at-risk students who could be targeted with a preventive intervention.

While previous research suggests guided interventions are more efficacious than unguided interventions, making the study of guided i-RFCBT our primary goal, clinically relevant benefits could still be obtained with unguided interventions as these have the potential to be accessed by larger numbers at a lower cost, and are essential for making highly scalable interventions. As a quasi-phase II pilot arm, we will therefore also include an unguided version of i-RFCBT to pilot its feasibility. This inclusion aims to assess the feasibility of retention and acceptability of the unguided intervention, as well as to estimate its effect sizes and variability to aid planning for a fully powered definitive trial of the unguided version, with respect to rates of incidence and levels of symptoms (descriptives and confidence intervals), as a separate analysis from the primary Phase III design.

## Methods/Design

### Study Design

#### Phase III efficacy study

The phase III study will consist of a single (researcher) blind parallel-group randomised-controlled trial (RCT), comparing guided i-RFCBT versus a no-intervention control group, with this our primary research question.

#### Quasi-Phase II pilot arm

A separate adjunct arm of unguided i-RFBCT will be included as a quasi-phase II pilot arm to enable a feasibility study of this intervention. This will be a separate question, and there will be no direct comparison between unguided i-RFCBT and the guided i-RFCBT or control arms in the phase III efficacy study.

### Setting

The study will be conducted over the internet and by telephone so we will recruit from around the UK. The intervention will be delivered on the internet, with the guided version being supported by trained staff at the University of Exeter.

### Participant inclusion criteria

Participants will be young adults resident in the UK aged 18 to 24 years and with elevated RNT, defined as scoring above the 75th percentile on at least one measure of worry/rumination (≥50 on the PSWQ [40] and/or ≥ 40 on the RRS [15]). Additionally, participants must be able to understand written English to engage with the intervention and have private internet access to ensure confidentiality. In line with standard practice, participants currently receiving antidepressant medication will be eligible, provided the dosage has been stable for at least the previous month.

### Participant exclusion criteria

Because this is a prevention study, participants will be excluded if they meet diagnostic criteria for a current (within past month) major depressive episode. Additionally, potential participants will be excluded if they report current and significant substance abuse or dependence; current symptoms of psychosis or bipolar disorder; current psychological therapy; or active suicide risk.

### Recruitment procedure

The main recruitment pathway involves contacting university departments around the UK by email and asking them to circulate an advert to their undergraduate students (as successfully used by Kingston et al. [48]). Generic discipline email addresses, or named individuals within the departmental administration team where possible, will be obtained using internet searches. Departments will be asked to circulate an included advert in the form of an attached pdf, containing a hyperlink to an online trial website that provides information and the initial online screening measures, to undergraduates within their department. The pdf format ensures departments can display the advert as a poster if they are not permitted to circulate by email. The number of emails sent out each week will be modulated depending on the response rates.

A Facebook and Twitter account will be set up to help circulate the advert, particularly to non-students. Relevant organisations working with young people and/or mental health issues will also be asked to advertise a link to the study on their website.

### Screening, baseline and consent procedure

#### Initial Online screening

An online screening website will be used to identify potentially eligible participants. A brief introduction to the study is included on the introductory page. Consent to online screening is obtained by informing potential participants that by clicking continue on this introductory page they are consenting for their responses to be stored by the researchers and that this data is subject to the Data Protection Act 1998.

A series of short questionnaires is then presented to identify potentially eligible participants by collecting basic demographic information (age, sex, ethnicity, location, and how they learned of the study) and screening on shortened versions of the PSWQ (four items, range 4 to 20, cut-off ≥ 12) and RRS (five items, range 5 to 20, cut-off ≥ 10) as used by the Dutch trial to identify individuals with elevated RNT (that is, > 75^th^ percentile) and the PHQ-8 [49] (PHQ-9 minus the suicidal thought question) to exclude participants who are likely to meet a diagnosis for current depression (cut-off ≥ 15).

Automated feedback informs participants whether they are eligible for further screening. Those excluded on low worry/rumination are informed that worry and rumination do not appear to be a significant problem for them and are thanked for their interest. Those excluded on high PHQ score are given a message explaining that their scores indicate low mood which is affecting their quality of life and that they may be experiencing an episode of clinical depression. This message advises them to see their GP and provides contact details of organisations offering support and advice. Eligible participants are invited to leave their contact details (name, telephone number and email address) as consent to be contacted. The full information sheet and consent form are then sent by email to the participant with an invitation to participate in a telephone interview with the researcher. The full information sheet (Additional file 1) and consent form (Additional file 2) are then sent by email to the participant with an invitation to participate in a telephone interview with the researcher.

#### Telephone screening and baseline assessment

The researcher will provide the interviewee with an overview of the trial, conduct the clinical interview and ask for verbal consent. Interviews will be audio-recorded, with the participant’s consent, so that the diagnostic status can be independently rated. The screening interview consists of brief screening questions on alcohol and drug use; symptoms of bipolar disorder and psychosis (Psychosis Screening Questionnaire, PSQ [50]); assessment of any relevant past or current treatments; and the Structured Clinical Interview for DSM-IV (SCID-I; [43]) sections on current and past depressive episodes, dysthymia and any relevant anxiety disorders and eating disorders. As the primary objective of this study is to investigate the prevention of depression, anxiety and eating disorders will be measured, but participants meeting the criteria for a disorder will not be excluded from this study.

Excluded participants are given feedback including advice to visit their GP and contact details of organisations for further support. Additionally, any risk reported during the interview is assessed using a well-established protocol to ensure that appropriate clinical support is obtained. Eligible participants are then asked to complete baseline measures, assessing episodic stress (the Episodic Life Event Interview, part of the UCLA Life Stress Interview [51]); levels of worry and rumination (PSWQ and RRS); symptom severity of depression and generalized anxiety (PHQ-9 and GAD-7); neuroticism (Eysenck Personality Questionnaire-Revised Neuroticism sub-scale (EPQ-R; [52]) and history (family history of depression, experience of abuse in childhood). With the exception of the episodic stress interview, which is always completed during the interview, baseline measures can be completed during the telephone interview, or returned by email/post.

#### Consent to trial

**Fig. 1:**
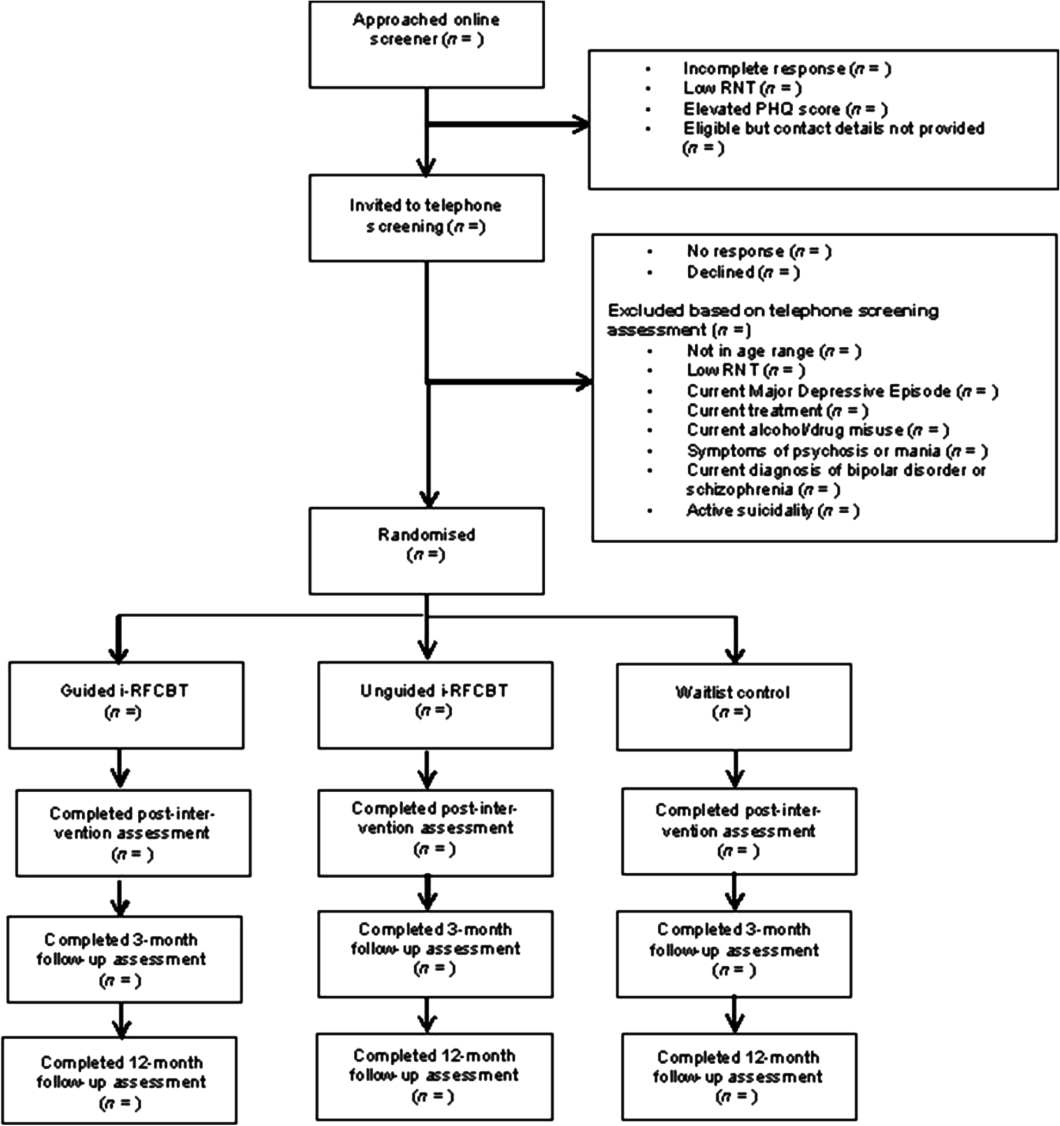
CONSORT diagram

All eligible participants will then be invited to enter the trial and asked to return the written consent to participate. Once consent is received, the participant will be randomised (see Fig. 1).

### Randomisation and allocation concealment

Independent computer-generated block randomisation to the guided i-RFCBT, unguided i-RFCBT or the no-intervention control group will be used. Randomisation will be stratified by sex and by past history of depression (presence or absence of past depressive episodes) to assess the effect of sex on outcomes and to examine potential differences between prevention of first onset and relapse prevention. The randomisation uses blocks of three to ensure close parity of randomisation across the intervention arms across time. Because of the two levels of stratification by sex and by level of depression, with blocks of three, the researcher would not be able to anticipate or determine allocation easily, and the use of varying block sizes was therefore deemed unnecessary. In order to preserve blinding of the study researcher, randomisation will be conducted by a third party not involved in assessing or treating the participants, and this third party will also inform the therapist, who will be responsible for informing participants of their allocation.

### Sample size calculation for phase III efficacy trial

Power and sample size calculations were based on testing the primary question of the efficacy of guided internet-RFCBT in terms of reducing the onset of major depression relative to the no-intervention control. For our primary comparison of guided i-RFCBT versus the no-intervention control, the Topper et al. study using the binary outcome of number of individuals meeting caseness for depression at 12 months, reported 13.1 % incidence of depression in guided i-RFCBT and 32.2 % incidence in the no-intervention control (hazard ratio = 0.41). Assuming similar effect sizes to the Topper et al. study for our intended time-to-outcome survival analysis over 12 months, then to detect the hazard ratio of 0.41 between these arms at the two-tailed 5 % alpha level, 75 participants must be recruited to each arm to provide 86 % power.

Estimates for change in depressive symptoms pre-to-post intervention for the guided i-RFCBT relative to the waiting list control indicate that 78 participants per arm provide a power of 80 % to detect the observed effect size of d = 0.51 from the Dutch trial at the two-tailed 5 % alpha level, allowing for the 20 % follow-up drop-out attrition observed in the Dutch study.

### Sample for quasi-phase II pilot arm

In the absence of any data on the unguided i-RFCBT, no power and sample size calculation was conducted for this arm. We will therefore aim to recruit the same number of participants for this arm as the other arms (n = 78), giving a total sample size of 234. The inclusion of this intervention arm is to explore the feasibility of this unguided intervention with respect to attrition, acceptability and estimates of incident rate and effect size.

### Interventions

#### Guided i-RFCBT

The guided intervention is an English version of i-RFCBT (called MindReSolve). RFCBT is derived from theoretical models [11] and experimental findings, which propose the existence of distinct types of repetitive thought (RT) with distinct consequences: constructive RT is characterized by a concrete, specific and action-oriented mode of processing, focusing on how events happen, whereas unconstructive RT is characterised by an abstract and evaluative processing mode, focusing on the meaning and implications of events and difficulties [11]. In experimental studies, relative to the abstract mode, the concrete mode improved problem-solving [53, 54] and reduced emotional reactivity in response to failure [55]. Underpinning RFCBT is the idea that shifting individuals into the concrete mode will reduce unconstructive rumination; consistent with this, repeated training of dysphoric and depressed participants to become more concrete reduces depression and rumination in both a proof-of-principle study and a randomised controlled trial [31, 33]. RFCBT therefore involves experiential and imagery exercises to adaptively shift processing mode (including concreteness, absorption and compassion), as well as functional analysis to help patients spot when rumination starts, distinguish between helpful versus unhelpful RT, and learn more functional responses. RFCBT seeks to change the process of thinking as opposed to the content of thoughts as in standard CBT [30]. In addition, rumination is conceptualised as a form of avoidance [11, 56], which is targeted on behavioural activation principles [57] with avoidance behaviour being replaced with more appropriate approach behaviours.

I-RFCBT consists of six modules, each taking around an hour to complete in session and 1 to 2 weeks to practise. It includes psycho-education, mood diaries, on-line experiential exercises using audio-recordings, pictures and video vignettes of students’ experiences of the therapy. The modules each follow the same basic structure: reflection on the previous session, introduction of a new technique, practical exercises and planning how to practise or implement the technique in daily life. The specific behaviour-change techniques are drawn from the following groups in the BCT Taxonomy (v1) [58]: goals and planning (goal setting, action planning, review behaviour and behavioural contract), feedback and monitoring (self-monitoring of behaviour and outcomes), shaping knowledge (information about antecedents), natural consequences (information about social and environmental consequences and monitoring of emotional consequences), associations (prompts/cues and associative learning), repetition and substitution (behavioural practice/rehearsal, behaviour substitution, and habit formation), antecedents (restructuring physical and social environment, avoiding/reducing cues for the behaviour), and self-belief (mental rehearsal of successful performance, focus on past success, and self-talk). The key strategies include coaching participants to spot warning signs for rumination and worry, and then to make IF-THEN plans in which an alternative strategy is repeatedly practised in response to the warning signs. These strategies include being more active, slowing things down, breaking tasks down, opposite action, relaxation, concrete thinking, becoming absorbed, self-compassion and assertiveness.

The intervention is accessed through a secure website, with each participant having a password protected account. Participants’ log-ins are automatically recorded by the programme, allowing for an automated measure of treatment compliance. Reminder emails will be sent to participants after 2 weeks if they have not completed the module.

The participant can work through each module at his own pace but can only move from one module to the next after the coach has provided feedback. The coach will provide feedback on these responses within 2 working days, in particular highlighting any positive steps made and encouraging participants to sustain these as well as pointing out areas to focus on over the next module. Participants will also be able to send questions to their assigned therapist throughout the programme if they are having difficulty with a specific exercise.

The intervention will be supported by qualified clinicians who have received specific training in the rumination-focused CBT approach. Treatment fidelity is ensured through the use of fixed modules in the platform, ensuring that all participants receive identical content. Detailed template responses for each module provide the coach with constrained feedback faithful with the treatment model, which they can then tailor to individual client responses. All responses from both the client and coach are automatically saved by the online platform, and a random sample will be checked against the templates to ensure they are faithful to the treatment. Furthermore, coaches will be provided with ongoing supervision with the developer of RFCBT to encourage fidelity. Supervision meetings will involve a brief overview of all clients and a more in-depth discussion of cases deemed to be more complex or where there is risk or non-response to the intervention.

#### Unguided i-RFCBT

The unguided version of the therapy contains the same six modules as the guided version, with almost identical content, adapted for self-help, including some conditional feedback on common difficulties with exercises. Participants are able to move freely from one module to the next, but are advised to spend 1 to 2 weeks on each to allow time for practice. Participants in the unguided version will be made aware that there is no coach monitoring their responses. However, they will be told that their questionnaire scores will be monitored on a weekly basis to check for any risk reported.

### Control condition

Participants in the control condition will be informed that they have been allocated to carry on as usual. In order to ensure participants’ welfare, participants are permitted to access any other treatments throughout the course of the study, as necessary. They will also be able to access the unguided i-RFCBT at the end of the study if they so wish.

### Blinding

This is a single blind study, with the researcher conducting outcome measures while blind to allocation. Participants will be asked at the end of the screening interview not to disclose their allocation to the researcher in any of their future correspondence with the researcher and reminded of the importance of this prior to and during each follow-up interview. Due to the intervention, participants and therapists cannot be blinded.

### Follow-up assessments and outcome measures

The primary outcome of interest of this study is the onset of a major depressive episode over a 12-month period, which we will assess with the SCID-I at 3 (post-intervention), 6 and 15 months after randomisation. The use of the SCID-I will allow for the ascertainment of depressive episodes that may have occurred between assessments (continuous time-to-onset and occurrence) and will also enable us to assess clinically significant symptoms of anxiety (particularly generalized anxiety disorder) that may have arisen in isolation or comorbid with depression. The severity of the depressive symptoms is measured using the PHQ-9 and of the anxiety symptoms using the GAD-7. The effect of the intervention on levels of worry and rumination will be assessed using the PSWQ and RRS. A number of potential confounding variables are also measured: stressful life events, using the Episodic Life Event Interview and any treatments received outside the trial (medication, therapy and use of self-help materials). Each assessment will take place via a telephone interview, with the option of the questionnaire measures being returned by email or post. To increase participant retention and completion of follow-ups, multiple attempts and multiple means (email, telephone and post) will be used to contact participants. Additionally, to increase motivation to complete the follow-up measures, lottery draws for £50 in vouchers will be held, with each participant receiving one ticket per completed follow-up.

### Feasibility and acceptability outcome measures (quasi-phase II pilot arm)

With respect to the unguided intervention, feasibility and acceptability of data collection procedures, levels of attrition, effect size and acceptability of the unguided internet RFCBT intervention will be measured to aid planning for a fully powered, definitive, phase III trial. The feasibility of data collection procedures will be assessed by measuring the missing items on the clinical outcome measures, the number and timing of drop-outs and whether these vary across arms. The acceptability of the intervention will be assessed using a behavioural index, which will measure the number of modules completed, the time spent logged into the site and which modules are completed more easily or frequently than others.

### Statistical analysis plan for phase III efficacy trial

Data cleaning will follow the protocol set out by Tabachnick and Fidell [59]. Statistical analysis will follow the CONSORT standards [60]. Unplanned missing data will be handled via multiple imputation (MI). Sensitivity analysis, assuming a variety of MI models (Missing at Random; Missing Not at Random), will verify the likely impact of missing data. Auxiliary variables will be used to improve the estimation of missing data. Primary analyses will be conducted on the intention-to-treat (ITT) sample.

Subsequent analyses will use the Complier Average Causal Effect (CACE) analysis [61, 62]. CACE assumes that randomisation has no direct effect on outcome variable; instead it assumes that the effect of treatment depends on the compliance to treatment (operationalized in terms of completing four out of six modules), which in turn is dependent on randomisation. Therefore, CACE provides estimates of a treatment effect whilst taking into account adherence and compliance with the treatment and whilst also retaining the benefits of randomisation. This model is thus an exemplar of using an Instrumental Variable (IV) where randomisation is the instrument, which is correlated with compliance to the treatment, and directly unrelated to the outcome. In simple terms, CACE finds the difference in the outcome variable between the compliers in the treatment arm and the compliers in the control arm had they been offered the treatment, assuming that the rates of compliance are similar in both arms as a consequence of randomisation.

As a prevention study, the main outcome of interest is the occurrence and time to onset of any depressive episode. Cox regression survival analyses will be performed to examine the effect of the preventive intervention on episode onsets of major depression. Participants will be censored upon measurement dropout or end of study. Although condition will be the main independent variable included in the model, we will also consider sex and history of past depression (the stratification variables).

The secondary outcome of occurrence/time to onset of generalized anxiety disorder will also be assessed using Cox regression survival analyses. Symptom severity and levels of rumination/worry will be examined using mixed model ANCOVAs: between group (ITT/CACE) and repeated measures (3 to 15-month follow-ups), with baseline symptom levels and stratification variables as covariates.

### Mediation and moderation analysis

Mediational analyses will be used to test the hypothesis that rumination acts as mediator of the treatment effects of condition on onset of major depression using the approach outlined by Kraemer et al. [63]. Potential moderators (for example, stratification variables and baseline characteristics) will also be investigated using this approach.

### Ethical approval

Ethical and professional guidelines will be followed at all times, in line with Good Clinical Practice guidelines. Ethical approval has been obtained from the Ethics Committee of the School of Psychology, University of Exeter (Ref: 2012/554). Any possible adverse events witnessed by the researcher or the therapist will be discussed as soon as possible with the supervisor. If this is deemed to be an adverse event, an adverse event report will be completed. In the case of serious adverse events, the University of Exeter, as sponsor, will also be notified using the same report and a follow-up phone call.

## Discussion

The current trial has been designed to build on the findings from the ZonMw trial, assessing the efficacy of guided i-RFCBT in a UK-based young adult population relative to a no-intervention control in the prevention of depression. Critically, to overcome the limitations of this study, diagnostic interviews at each assessment point will allow for more accurate measures of incidence rates across the course of the follow-up period.

I-RFCBT may provide an effective and acceptable intervention for the prevention of depression in young adults. Widespread implementation is a key factor in prevention and the internet may provide a valuable tool in increasing access. Furthermore, while previous evidence suggests guided therapy will be more efficacious, the assessment of an unguided version allows a preliminary investigation of the potential benefits of increased availability and reduced cost that this may provide.

## Trial status

Recruitment began in November 2013 and was ongoing at the time of submission.

## Additional files

Additional file 1:
**Participant information sheet.** (PDF 178 kb) Additional file 2:
**Consent form.** (DOCX 17 kb)

## Abbreviations

CONSORT: Consolidated Standards of Reporting Trials
EPQ-R: Eysenck Personality Questionnaire-Revised Neuroticism sub-scale
GAD-7: Generalized anxiety disorder 7-item scale
GP: general practitioner
ITT: intention-to-treat
MI: multiple imputation
MRC: Medical Research Council
PHQ-8/PHQ-9: Patient health questionnaire-8 item or 9 item
PSQ: Psychosis Screening Questionnaire
PSWQ: Penn State Worry Questionnaire
RCT: randomised controlled trial
RFCBT: rumination-focused cognitive-behavioural therapy
RNT: repetitive negative thought
RRS: Ruminative Response Scale of the Response Styles Questionnaire
RT: repetitive thought
SCID-I: Structured Clinical Interview for DSM-IV
TAU: treatment-as-usual
