# Supplementary material for: Guided, internet-based, rumination-focused cognitive behavioural therapy (i-RFCBT) versus a no-intervention control to prevent depression in high-ruminating young adults, along with an adjunct assessment of the feasibility of unguided i-RFCBT, in the REducing Stress and Preventing Depression trial (RESPOND): study protocol for a phase III randomised controlled trial
Source: Trials. 2016 Jan 4;17:1. doi: 10.1186/s13063-015-1128-9 (PMC4698823; doi:10.1186/s13063-015-1128-9)
Supplement: Additional file 2: — Consent form. (DOCX 17 kb) [file 13063_2015_1128_MOESM2_ESM.docx]

**Consent Form**

**Study Title: Targeting worry and rumination to prevent the onset of depression in young adults:** A randomised-controlled trial comparing guided and unguided internet-based rumination focused cognitive behavioural therapy and a no treatment control.

**Researchers:** Lorna Cook, PhD student, supervised by Prof. Ed Watkins

Please write/type your initials in the boxes below if you agree with the following statements:

| 1. I have read and understood the information sheet provided and have had the opportunity to ask further questions. |  |
| --- | --- |
| 1. I understand that my GP retains clinical responsibility for me throughout the study and will be contacted in the event of any significant risk of harm to myself or others. |  |
| 1. I understand that my participation in the study is voluntary and that I may withdraw from the study at any time, without giving any reason for doing so. |  |
| 1. I give consent for my telephone interviews to be audio-recorded so that the research team can check the conduct and reliability of these interviews. (Note: You may still participate without consenting to this audio-recording) |  |
| 1. I give my informed consent to participate in this study. |  |

Participant Name (please print):

Participant email address:

Participant telephone number:

Participant signature: Date:

Researcher Name: Date:

Researcher Signature:

If you are willing to be contacted about participating in further research at the Mood Disorders Centre, please write your initials in the box below. If you do not wish to be contacted, simply leave this box blank.

| I am willing to be contacted about participating in further research at the Mood Disorders Centre. |  |
| --- | --- |

**Please return the completed consent form to Lorna Cook by email (**[**lzc204@exeter.ac.uk**](mailto:lzc204@exeter.ac.uk)**) or by post: Sir Henry Wellcome Building for Mood Disorders Research, University of Exeter, Perry Road, EX4 4QG**

**You will receive a copy signed by the researcher for your records.**
